# Tctp regulates the level and localization of Foxo for cell growth in *Drosophila*

**DOI:** 10.1038/s41420-022-00937-2

**Published:** 2022-03-31

**Authors:** Sujin Nam, Thao Phuong Le, SeYeon Chung, Kwang-Wook Choi

**Affiliations:** 1 Department of Biological Sciences, Korea Advanced Institute of Science & Technology, 291 Daehak-ro, Yuseong-gu, Daejeon 34141 Korea; 2 Department of Biological Sciences, Louisiana State University, Baton Rouge, LA 70803 USA

**Keywords:** Development, Cell biology

## Abstract

Regulation of cell size is crucial for organ development. Insulin signaling regulates organ size by antagonizing the subgroup O of forkhead box transcription factor (Foxo) through 14-3-3 in *Drosophila*. However, mechanisms for controlling the level and the nuclear localization of Foxo in developing organs are not well understood. Here, we investigate the role of *Drosophila* Translationally controlled tumor protein (Tctp) and its interacting partner 14-3-3 in Foxo regulation during organ development. Foxo overexpression in the developing eye disc results in growth inhibition. We show that Tctp overexpression antagonizes the Foxo effect by downregulating the Foxo level in the eye disc. Foxo overexpression or knockdown of Tctp in the larval salivary gland results in reduced gland size, mainly due to reduced cell size by defects in endoreplication. Whereas *14-3-3ζ* knockdown has a negligible effect, knockdown of *14-3-3ε* mimics the effect of Foxo overexpression or *Tctp* knockdown, suggesting an isoform-specific role of 14-3-3. Unlike nuclear enrichment of the endogenous Foxo in the salivary gland, overexpressed Foxo protein is largely distributed in the cytoplasm, and this mislocalization is restored by Tctp overexpression. Opposite to the effect of Tctp overexpression, *Tctp* knockdown increases cytoplasmic Foxo levels while decreasing nuclear Foxo levels. Together, our data suggest that Tctp and 14-3-3ε play critical roles in cell growth by reducing cytoplasmic Foxo levels. Knockdown of human TCTP also elevates the level of cytoplasmic FOXO1 in HeLa cells, suggesting that human TCTP may have a conserved role in downregulating FOXO in human cells.

## Introduction

Translationally controlled tumor protein (TCTP) is a family of conserved proteins that play diverse functions ranging from protein synthesis to allergic responses [1]. TCTP is also important for growth control and has been implicated in tumorigenesis and tumor reversion [2, 3].

*Drosophila* TCTP (labeled Tctp) is essential for organ growth by regulating both cell size and number in imaginal discs, the primordia for adult organs. Studies in *Drosophila* and *Arabidopsis* have revealed the roles of TCTP in the Target of rapamycin (TOR) signaling pathway for tissue growth [4–6]. Our previous study has shown a critical role of 14-3-3 in promoting TOR signaling along with Tctp [7]. 14-3-3 proteins are adapter molecules that modulate protein functions in diverse signaling pathways. Two homologous 14-3-3 proteins in *Drosophila*, 14-3-3ε and 14-3-3ζ, are functionally redundant in organ development [8, 9].

14-3-3 proteins have additional functions in insulin signaling for retinal differentiation and aging in *Drosophila*, where they are involved in the negative regulation of the Foxo transcription factor [10]. In the absence of insulin signaling, Foxo activity is increased to inhibit retinal development, resulting in the reduction and roughness of the adult eye. In contrast, increased insulin signaling leads to an inactivation of the Foxo function through 14-3-3. Mammalian FOXO proteins are phosphorylated by Akt upon insulin signaling [11–13] (Hereafter, *Drosophila* and mammalian proteins are labeled as Foxo and FOXO, respectively). Phosphorylated FOXO proteins are excluded from the nucleus, thus resulting in reduced nuclear FOXO function as a transcription factor [14]. These studies suggest that the inhibitory effects of Foxo overexpression in the *Drosophila* eye might be related to an activation of the nuclear Foxo function. However, it remains unclear whether the effects of Foxo overexpression are solely dependent on the nuclear function of Foxo in vivo.

The roles of Tctp and 14-3-3 in promoting the growth of imaginal discs [7] raise the possibility that Tctp might also be linked to the Foxo function. The antagonistic relationship between 14-3-3 and FOXO [10, 14–16] led us to hypothesize that growth defects in the imaginal discs by loss of Tctp or 14-3-3 isoforms may be in part due to an activation of the nuclear Foxo function. Accordingly, loss of Tctp or 14-3-3 may promote the nuclear localization of Foxo.

In this study, we tested this hypothesis to determine the functional relationship between Tctp, 14-3-3, and Foxo in organ development. We provide evidence that Tctp and 14-3-3ε negatively regulate Foxo to promote normal cell growth in imaginal discs and larval salivary glands. Unexpectedly, our data show that Tctp and 14-3-3ε are required to inhibit Foxo accumulation in the cytoplasm. This study suggests that the inhibitory role of Foxo in cell growth is correlated with its cytoplasmic accumulation rather than its nuclear function.

## Results

### Effects of Foxo overexpression on eye growth are antagonized by Tctp

Foxo overexpression by *GMR-Gal4* in differentiating retinal cells reduces the eye size. 14-3-3 overexpression suppresses this Foxo overexpression phenotype [10]. We have previously shown that loss of Tctp and 14-3-3 synergistically reduces the eye [7]. These genetic interactions led us to examine how Tctp and 14-3-3 are related to the Foxo function in organ development.

**Fig. 1:**
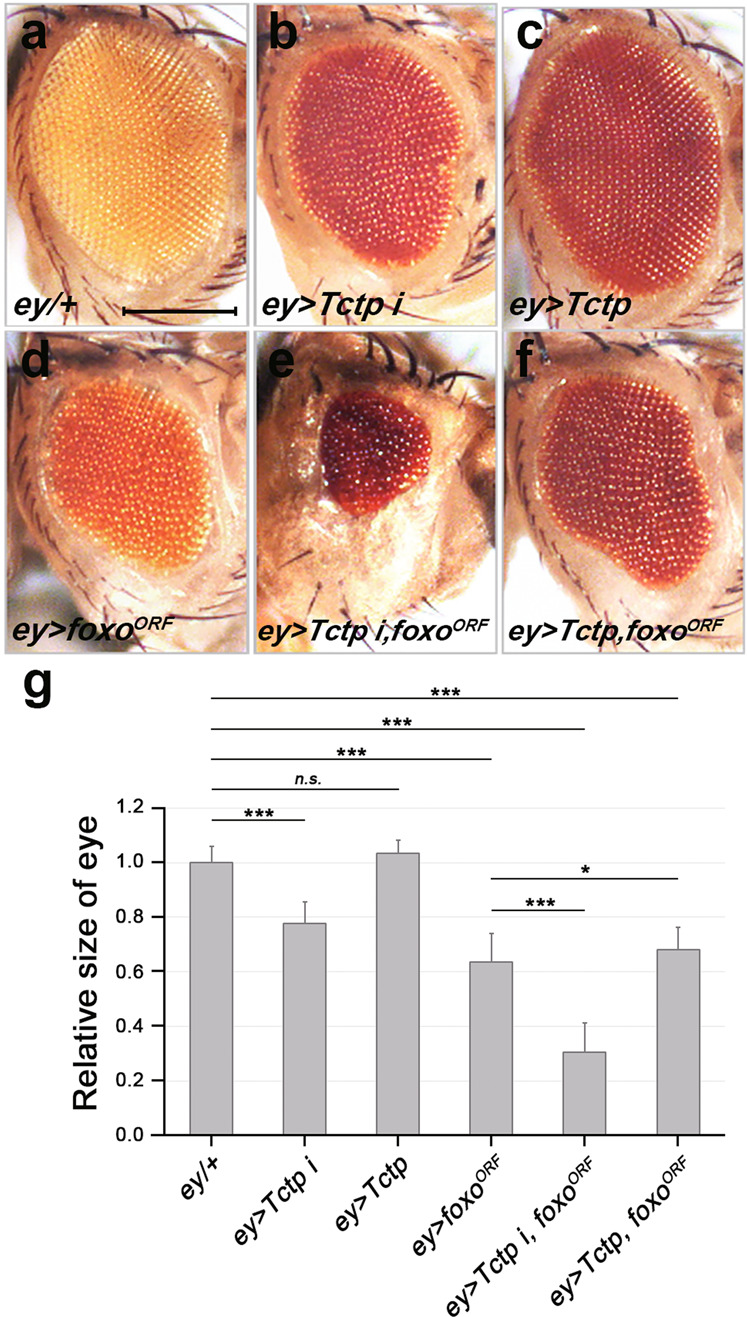
Tctp and Foxo show an antagonistic relationship in eye development. **a**–**f** The size of adult eyes. **a** Control *ey-Gal4/+* shows normal eye (*n* = 49). **b** *ey* > *Tctp RNAi* shows a weak reduction in eye size (*ey* > *Tctp i* in short. *n* = 24). **c** *ey* > *Tctp* (*n* = 14) has normal eye. **d** *ey* > *foxo*^*ORF*^ (*n* = 33) shows an eye size reduction. **e** *ey* > *Tctp i, foxo*^*ORF*^ (*n* = 15) shows severe eye size reduction. **f** *ey* > *Tctp, foxo*^*ORF*^ (*n* = 37). Tctp overexpression weakly suppresses the Foxo overexpression phenotype. **g** Quantification of eye sizes shown in (**a**–**f**). The areas of eyes were measured using the Image J program. The eye sizes relative to the control are presented with standard deviation indicated by error bars. Their statistical relationships are indicated by black asterisks (*p < 0.05, **p < 0.01, ***p < 0.001, and *n.s*., not significant). Scale bar, 100 μm.

Because Tctp is mainly required in undifferentiated cells of the eye disc, we used *eyeless (ey)-Gal4* instead of *GMR-Gal4* to drive overexpression or silencing of genes in proliferating cells anterior to the morphogenetic furrow (MF). Compared to control (*ey-Gal4/+*) (Fig. 1a), *Tctp* RNAi caused a ~20% reduction of the eye size (Fig. 1b, g), whereas Tctp overexpression did not affect the eye size (Fig. 1c, g). Overexpression of Foxo using *foxo*^*UAS.ORF.GW.Tag:HA*^ (*foxo*^*ORF*^) resulted in a ~40% reduction of the eye size (Fig. 1d, g). *Tctp* RNAi with *foxo*^*ORF*^ overexpression led to a ~70% eye size reduction (Fig. 1e, g). This strong enhancement of the reduced eye size phenotype raised the possibility of an antagonistic interaction between *Tctp* RNAi and *foxo*^*ORF*^ overexpression. Alternatively, it may be an additive effect of these two conditions. To distinguish these possibilities, we examined whether Tctp overexpression suppresses the effects of Foxo overexpression. Whereas Tctp overexpression alone did not affect the eye size in the wild-type background (Fig. 1c, g), it slightly increased the size of Foxo-overexpressing eyes (Fig. 1f, g). Hence, Tctp may antagonize the Foxo effects in eye development.

**Fig. 2:**
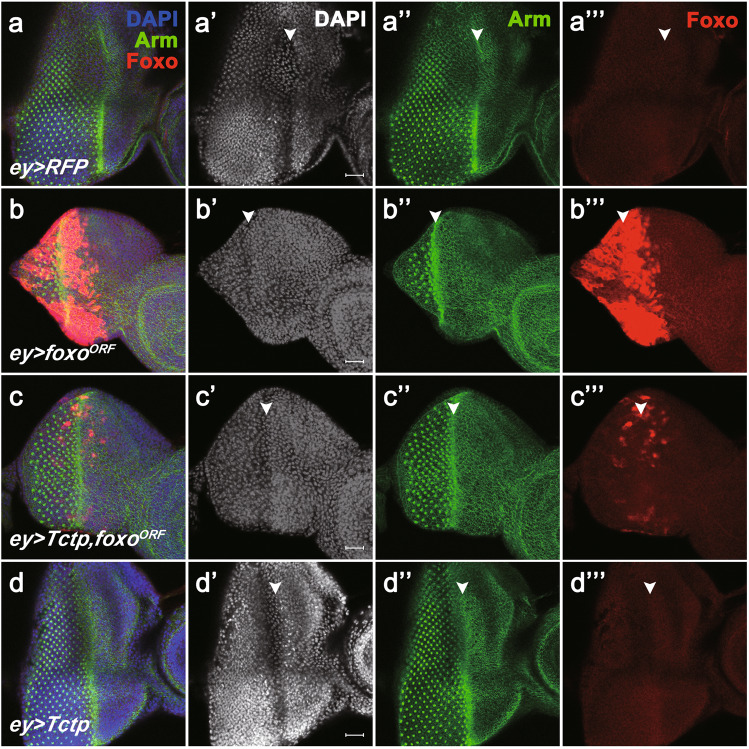
Tctp negatively regulates ectopic Foxo. **a**–**d** Eye/antenna discs from 3^rd^ instar larvae were stained with DAPI, anti-Arm, and anti-Foxo antibodies as indicated. **a**–**a'''** *ey* > *RFP* control shows normal arrays of ommatidial clusters labeled by Arm staining posterior to the morphogenetic furrow (MF, white arrowheads) (**a''**). Foxo is ubiquitously expressed at a low level and is slightly elevated along the furrow. **b**–**b'''** *ey* > *foxo*^*ORF*^. Foxo overexpression causes a considerable reduction in the eye disc size and the number of photoreceptor clusters (**b''**). The disc shows a high level of Foxo staining due to *foxo*^*ORF*^ overexpression (**b'''**). **c**–**c'''** *ey* > *Tctp, foxo*^*ORF*^. The inhibition of photoreceptor differentiation by *foxo*^*ORF*^ overexpression is partially suppressed by Tctp co-expression, and Foxo levels are reduced by Tctp overexpression in 70% of eye discs examined (*n* = 27). **d–d'''** *ey* > *Tctp* shows no significant differences to control. Anterior is to the right. Scale bar, 20 μm.

**Fig. 3:**
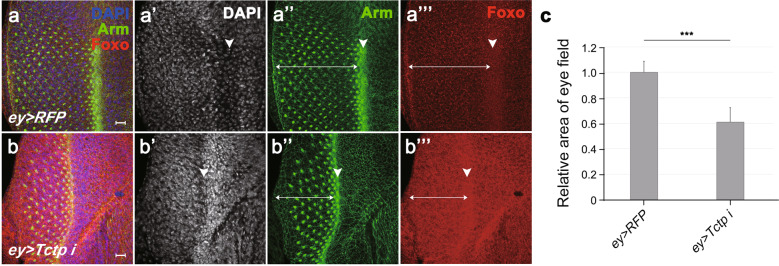
Knockdown of Tctp increases endogenous Foxo levels in the eye disc. **a**–**b** Eye discs from third instar larvae were stained with DAPI and antibodies for Arm and Foxo. **a**–**a'''** *ey* > *RFP* control. The eye field with photoreceptor clusters is indicated by an arrow, and the morphogenic furrow is marked by an arrowhead. **b**–**b'''** *ey* > *Tctp i*. *Tctp* RNAi reduces the size of the eye field as indicated by fewer ommatidial arrays (**b''**) compared with the control (**a''**). Foxo staining is increased in *Tctp* RNAi discs. Scale bar, 10 μm. **c** Quantification of the relative eye field based on the column number of photoreceptor clusters. (*n* = 8 discs for *ey* > *+* and 7 discs for *ey* > *Tctp RNAi*).

We further examined the effects of Tctp overexpression on the Foxo expression pattern in the larval eye disc. Immunostaining of the wild-type disc showed weak Foxo signals distributed in the entire eye disc (Fig. 2a–a'''). Immunostaining for the adherens junction marker Armadillo (Arm) [17] showed regular arrays of differentiating ommatidia posterior to the morphogenetic furrow (Fig. 2a''). *foxo*^*ORF*^ overexpression by *ey-Gal4* strongly increased Foxo level in the anterior to the furrow, with weaker Foxo signals in the posterior region (Fig. 2b–b'''). Eye discs with Foxo overexpression were reduced in size and showed only a few columns of photoreceptor clusters (Fig. 2b''). When *Tctp* and *foxo*^*ORF*^ were co-overexpressed, the exogenous Foxo level was considerably decreased (Fig. 2c–c'''). The decreased Foxo levels were correlated with partial suppression of the retinal defects by *foxo*^*ORF*^ overexpression (Fig. 2c''). Tctp overexpression alone did not affect the low levels of endogenous Foxo (Fig. 2d'''). However, *Tctp* RNAi significantly increased endogenous Foxo level compared to control discs expressing red fluorescent protein (RFP) (Fig. 3a–b''') and reduced the size of the eye field by 40% compared to control (Fig. 3b, c). These results suggest that Tctp negatively regulates the level of Foxo in the developing eye disc.

### Tctp and 14-3-3ε are required for cell growth in salivary glands

**Fig. 4:**
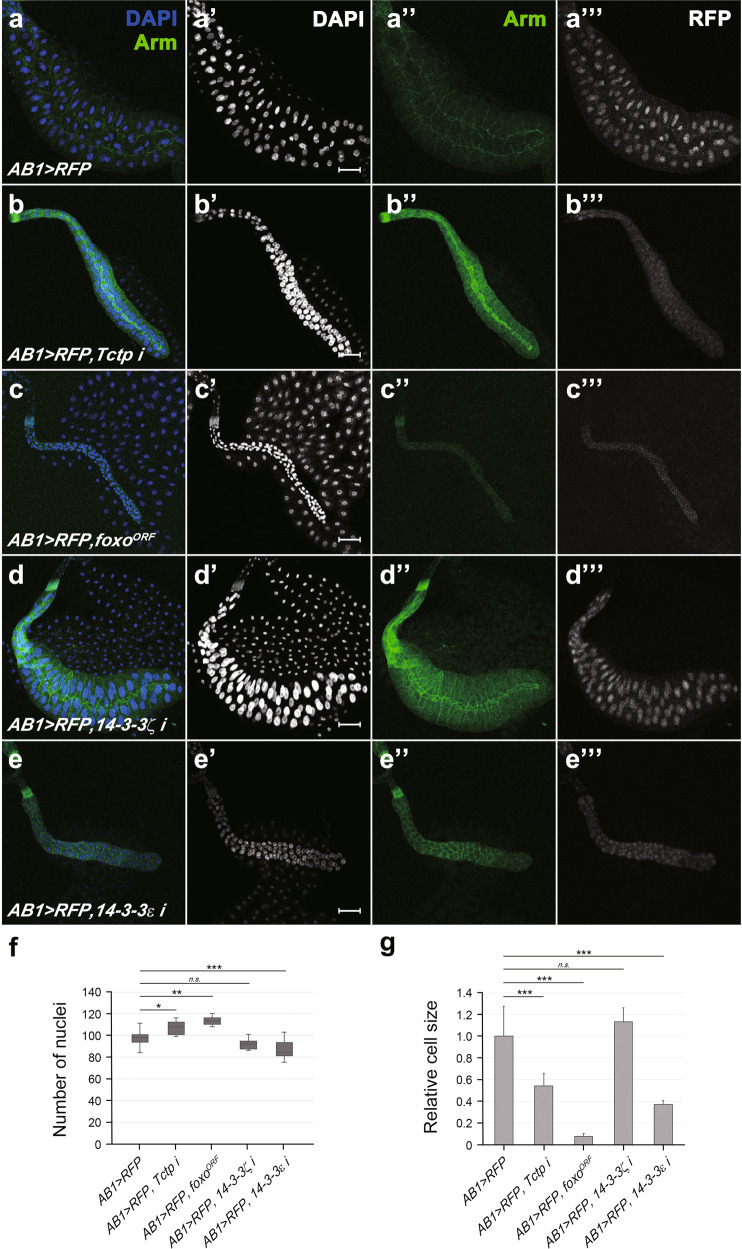
*Tctp* RNAi, *14-3-3ε* RNAi, or Foxo overexpression reduces salivary gland size by affecting cell size. **a**–**e** Salivary glands of late 3^rd^ instar larvae were stained with DAPI and antibodies for Arm. **a**–**a'''** *AB1* > *RFP* control. The gland is normal in size and the pattern of Arm staining (**a''**) and RFP (**a'''**) expression. **b**–**b'''** *AB1* > *RFP, Tctp i. Tctp* knockdown shows a thin salivary gland. **c**–**c'''** *AB1* > *RFP, foxo*^*ORF*^. Foxo overexpression induces a striking reduction of tissue and nucleus size in the salivary gland. **d**–**d'''** *AB1* > *RFP,14-3-3ζ i*. Knockdown of *14-3-3ζ* has negligible effect on salivary gland development. **e**–**e'''** *AB1* > *RFP, 14-3-3ε i. 14-3-3ε RNAi* leads to a strong reduction in gland size. **f** Quantification of cell numbers based on the counting of the DAPI-stained nuclei. *AB1* > *RFP*, *n* = 18; *AB1* > *RFP, Tctp i*, *n* = 7; *AB1* > *RFP, foxo*^*ORF*^, *n* = 4; *AB1* > *RFP, 14-3-3ζ i*, *n* = 10; *AB1* > *RFP, 14-3-3ε i*, *n* = 16 glands. **g** The relative cell size from cell boundaries stained with anti-Arm antibody. Scale bar, 100 μm in **a'**-**e'**; Error bars in (g) indicate standard deviation (SD); *AB1* > *RFP*, *n* = 17 cells from three glands; *AB1* > *RFP, Tctp i*, *n* = 12 from four glands; *AB1* > *RFP, foxo*^*ORF*^, *n* = 23 from four glands; *AB1* > *RFP, 14-3-3ζ i*, *n* = 6 from two glands; *AB1* > *RFP, 14-3-3ε i*, *n* = 12 from four glands; ***<0.001; **<0.01; *<0.05; *n.s*. (not significant); *N* = 2.

Reduced eye disc size by Foxo overexpression suggests that Foxo affects cell proliferation during eye development. Tctp regulates cell size as well as cell proliferation in the wing [18]. To test whether Foxo overexpression also affects cell size, we examined the larval salivary gland, a useful organ for studying cell growth and morphogenesis due to its large cell size [19]. *Tctp* knockdown in the salivary gland by two independent *UAS-Tctp RNAi* lines using the *AB1-Gal4* driver [20] caused a similar reduction in the gland size (Fig. 4b–b''' and Supplementary Fig. 1b-c, h). Furthermore, Tctp overexpression partially restored the salivary gland size (Supplementary Fig. 2g, h). We quantified cell numbers and cell size by counting DAPI-stained nuclei and measuring areas based on Arm signals along cell junctions, respectively (Fig. 4f, g). Whereas *Tctp* RNAi only slightly increased the cell number (9%, *n* = 7; Fig. 4f), *Tctp* RNAi strongly reduced the cell size (54 ± 12% of wild-type size, *n* = 12 from four glands; Fig. 4g). Hence, the major effect of *Tctp* knockdown appears to be an inhibition of cell growth, although it may have minor effects on cell number. Overexpression of *foxo*^*ORF*^ also severely reduced the gland size (Fig. 4c–c'''). The cell size was strongly reduced (8 ± 3% of the wild-type size, *n* = 23 from four glands; Fig. 4g), although the cell numbers were slightly increased (16%, *n* = 4; Fig. 4f).

Based on the negative regulation of Foxo by 14-3-3 [10], we tested whether knockdown of 14-3-3 impairs the growth of salivary glands as seen with Foxo overexpression. Because two isoforms, 14-3-3ε and 14-3-3ζ, have redundant functions in imaginal discs, a single knockdown of either *14-3-3ε* or *14-3-3ζ* does not affect the development of imaginal discs [7, 9]. As expected, *14-3-3ζ* RNAi (v48725) did not considerably affect salivary gland development (Fig. 4d–d''', f, g). Two other independent *14-3-3ζ* RNAi lines (BDSC 28327 and 41878) showed similar results (Supplementary Fig. 1f-g, h). Unexpectedly, however, *14-3-3ε* RNAi (31196R-3), which has been used in other studies [7, 21, 22], resulted in a strong reduction of the salivary gland size (Fig. 4e–e''') as seen with *foxo*^*ORF*^ overexpression (Fig. 4c–c'''). Another *14-3-3ε* RNAi line (BDSC34884) also showed a weaker but significant reduction of the salivary gland size (Supplementary Fig. 1d, h). Interestingly, 14-3-3ε overexpression showed a weak but significant negative effect on gland development (Supplementary Fig. 2c, h), suggesting that 14-3-3ε must be tightly regulated for proper growth of the gland. Perhaps due to such adverse effect of 14-3-3ε overexpression, growth defects of *14-3-3ε* RNAi were not restored by 14-3-3ε overexpression (Supplementary Fig. 2d, h). Growth defects by *14-3-3ε* RNAi alone suggest that loss of *14-3-3ζ* may be compensated by 14-3-3ε, whereas 14-3-3ε has an essential role for gland development that cannot be replaced by 14-3-3ζ. *14-3-3ε* RNAi slightly reduced the cell number (11%, *n* = 16; Fig. 4f), but strongly reduced the cell size (37 ± 4% of wild-type size, *n* = 12 from four glands; Fig. 4g). Hence, like Tctp, 14-3-3ε seems to be required mainly for controlling cell size.

### Loss of Tctp or 14-3-3ε impairs endoreplication in the larval salivary gland

**Fig. 5:**
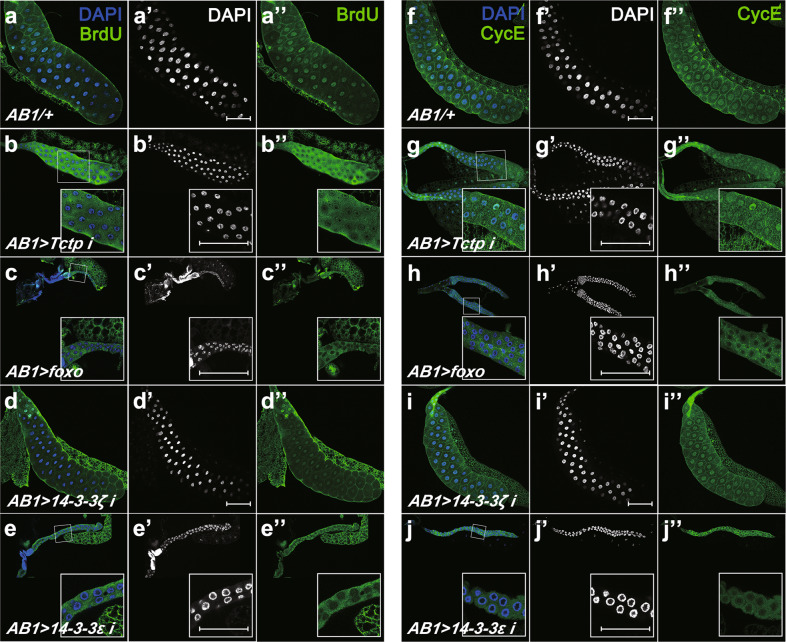
Tctp, Foxo, or 14-3-3ε affects endoreplication in larval salivary glands. **a**–**e** Salivary glands are labeled with DAPI and BrdU (DNA replication marker). **a**–**a''** Control salivary gland cells show high levels of BrdU in the nuclei. **b**–**b''** Compared to control, *Tctp* RNAi leads to a lower level of BrdU in the nuclei. **c**–**c''** Overexpressing Foxo results in a small salivary gland. BrdU is strongly reduced in the nuclei. **d**–**d''** 14-3-3ζ knockdown does not affect the gland size or the BrdU levels in the nuclei. **e**–**e''** *14-3-3ε* RNAi causes a strong reduction in gland size and nuclear BrdU staining. **f–j** Salivary glands are immunostained with anti-CycE and DAPI. **f**–**f''** The control gland shows high levels of CycE in the nuclei. **g**–**g''** Salivary glands with *Tctp* RNAi show CycE mislocalized to the cytoplasm. **h**–**h''** Overexpression of Foxo results in severe loss of nuclear CycE. **i**–**i''** The reduction of 14-3-3ζ does not affect the nuclear localization of CycE. **j**–**j''** Knockdown of 14-3-3ε leads to the near absence of CycE in the nuclei. The white box at the bottom right of each panel is a magnification of the small white box in the same panel. Scale bars, 150 μm (**a'**, **b'**, **d'**, **f'**, **g'**, and **i'**), 75 µm (**c'**, **e'**, **h'**, and **j**).

The larval salivary gland grows primarily by endoreplication, resulting in polyploidy cells through multiple S phase replications without entering mitosis [23, 24]. Therefore, we tested whether Tctp and 14-3-3ε are required for proper endoreplication by checking the level of bromodeoxyuridine (BrdU) incorporation. In the control gland (*AB1/*+), BrdU signal was enriched in cell nuclei (Fig. 5a-a''). In contrast, *Tctp* RNAi resulted in significantly reduced BrdU signals in cell nuclei (Fig. 5b-b''). A similar reduction of nuclear BrdU signals was seen with Foxo overexpression (Fig. 5c-c''). While *14-3-3ζ* RNAi showed nuclear BrdU signals similar to control (Fig. 5d-d''), *14-3-3ε* RNAi led to a significant decrease in nuclear BrdU signals (Fig. 5e-e''). Loss of nuclear BrdU signals by *Tctp* RNAi*, 14-3-3ε* RNAi or Foxo overexpression was consistent with the reduced sizes of salivary glands (Fig. 4).

Our previous work has shown that Tctp is required for normal levels of Cyclin E (CycE) in imaginal discs [18]. Oscillation of CycE expression is critical for repeated cycles of endoreplication in salivary gland cells [25]. Hence, we checked whether reduced nuclear BrdU signals by *Tctp* RNAi are related to defective CycE regulation. Control glands showed CycE expression preferentially localized in the nucleus (Fig. 5f''). *Tctp* RNAi reduced nuclear CycE levels while increasing cytoplasmic CycE staining, especially in the distal part of the salivary gland (Fig. 5g''). *14-3-3ε* RNAi or Foxo overexpression resulted in a more pronounced loss of nuclear CycE and a gain of cytoplasmic CycE (Fig. 5h'' and j''). In contrast, *14-3-3ζ* RNAi showed a normal pattern of CycE expression (Fig. 5i''). These results suggest that Tctp and 14-3-3ε are necessary for CycE-dependent endoreplication in larval salivary glands.

### Tctp overexpression downregulates cytoplasmic Foxo levels

**Fig. 6:**
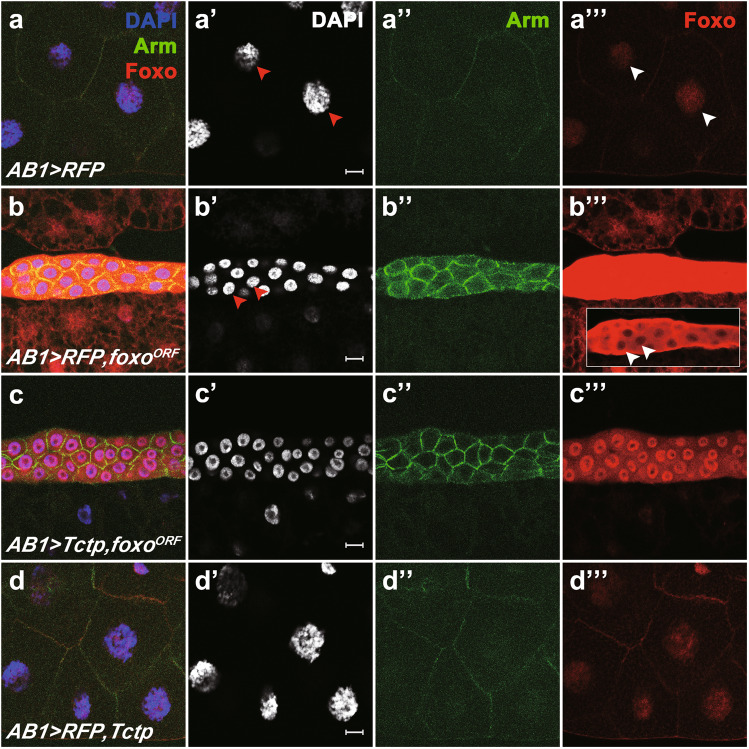
Tctp promotes nuclear localization of Foxo in salivary glands. Effects of Tctp on Foxo level and localization. **a**–**d** Salivary glands from late third instar larvae were stained with DAPI and antibodies for Arm and Foxo. **a**–**a'''** *AB1* > *RFP*. It shows a control level of the adherent junction marker Arm (**a''**) and Foxo (**a'''**). Foxo is enriched in the nucleus (arrowheads; red in **a’** and white in **a'''**). **b**–**b'''** *AB1* > *RFP, foxo*^*ORF*^. The size of salivary glands is greatly reduced (**b**). Sizes of nuclei (**b'**) and cells (**b''**) are reduced by Foxo overexpression (**b'''**). Arm staining is stronger than the control level. The same gland at a lower (20%) laser intensity shows that Foxo staining is mainly cytoplasmic (inset in **b'''**). **c**–**c'''** *AB1* > *Tctp, foxo*^*ORF*^. Tctp overexpression reduces the level of ectopic Foxo and results in the nuclear localization of Foxo (**c'''**). However, the cell size was not fully rescued. **d**–**d'''** *AB1* > *RFP, Tctp*. Tctp overexpression alone has little effect in normal salivary glands. Scale bar, 20 μm.

The function of Foxo as a transcription factor has been implicated in cell growth inhibition [26, 27]. Hence, we expected that Foxo overexpression would increase the level of Foxo in the nucleus, thus reducing cell size. In control glands expressing RFP, endogenous Foxo was preferentially localized in the nucleus at low levels (Fig. 6a'''). Overexpression of *foxo*^*ORF*^ severely reduced the gland size (Fig. 6b), with a saturated level of intense Foxo signals (Fig. 6b'''). Unexpectedly, confocal imaging at a lower laser intensity showed that overexpressed Foxo was enriched in the cytoplasm rather than the nucleus (Inset in Fig. 6b'''). Tctp overexpression alone did not affect the Foxo localization (Fig. 6d'''). However, when Tctp and Foxo were co-overexpressed, exogenous cytoplasmic Foxo level was significantly decreased while nuclear Foxo level was increased (Fig. 6c'''). Despite the reduction of cytoplasmic Foxo, Tctp overexpression was insufficient to restore the reduced gland size caused by Foxo overexpression, which may be due to high Foxo levels remaining in the cytoplasm and nucleus (Fig. 6c'''). In contrast to the effect of Tctp overexpression on Foxo distribution, 14-3-3ε overexpression did not suppress the Foxo overexpression phenotype (Supplementary Fig. 3c–d'), which might be due to the negative effect of 14-3-3ε overexpression (Supplementary Fig. 2c).

### Tctp and 14-3-3ε are required to suppress cytoplasmic Foxo levels

Our data shown above indicated that Tctp overexpression reduces the level of ectopic Foxo expressed from *foxo*^*ORF*^ overexpression (Figs. 2 and 6). These results led us to test whether Tctp affects the level of endogenous Foxo in the salivary gland. Endogenous Foxo was enriched in the nucleus, with low levels in the cytoplasm and cell membranes (Supplementary Fig. 4a'''). When *Tctp* was knocked down, cytoplasmic Foxo was increased while nuclear Foxo was reduced (Supplementary Fig. 4b'''). Overexpression of Tctp in the *Tctp* RNAi condition led to a significant recovery of Foxo in the nucleus (Supplementary Fig. 4c'''-c''''). These data suggest that Tctp is required for nuclear localization of Foxo and its downregulation in the cytoplasm.

Because *Tctp* RNAi and *14-3-3ε* RNAi resulted in similar phenotypes in the salivary gland, we examined whether these two genes are functionally related. First, we tested whether loss of *14-3-3ε* affects the subcellular Foxo localization. As seen with *Tctp* RNAi, *14-3-3ε* RNAi increased the cytoplasmic Foxo level, but *14-3-3ζ* RNAi did not (Supplementary Fig. 5b''', c'''). Next, we examined the combined effects of *Tctp* RNAi and *14-3-3* RNAi. Salivary glands depleted in both *14-3-3ε* and *Tctp* showed a similar size reduction to *14-3-3ε* RNAi alone (Supplementary Fig. 6b). The lack of additional reduction by double knockdown suggests that Tctp and 14-3-3ε may be required for the nuclear localization of Foxo in the same pathway. Interestingly, although *14-3-3ζ* RNAi caused no detectable defects in the Foxo pattern (Supplementary Fig. 5c'''), it partially suppressed the effects of *Tctp* RNAi, resulting in the recovery of nuclear Foxo (Supplementary Fig. 6c''').

### Cytoplasmic human FOXO1 is upregulated by knockdown of TCTP or YWHAE

**Fig. 7:**
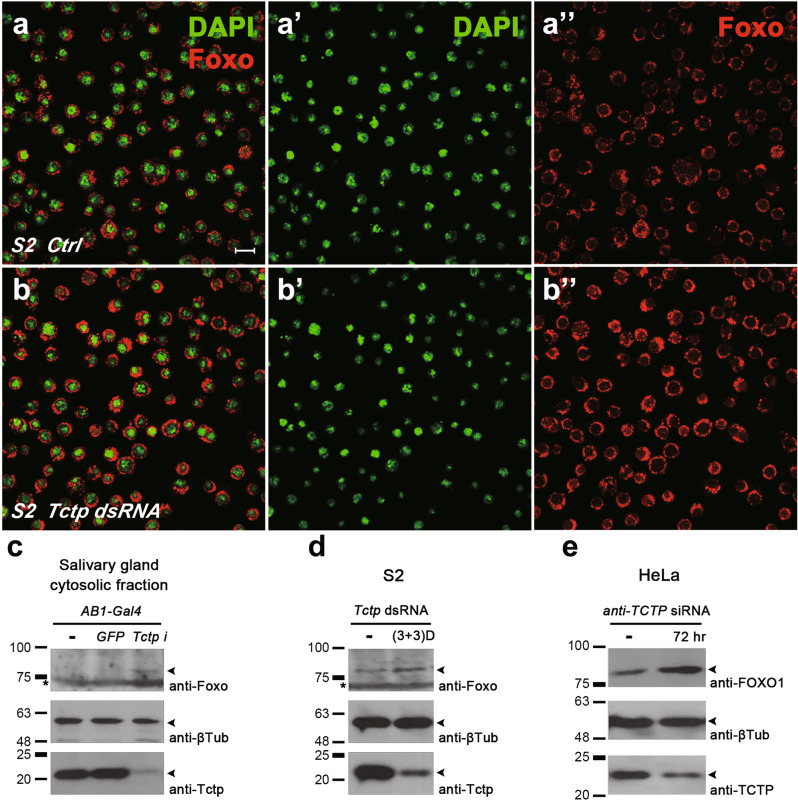
Endogenous Foxo is enriched in the cytoplasm by Tctp knockdown. **a**–**b** Immunocytochemical effects of Tctp knockdown on Foxo levels. S2 cells were stained with DAPI and the anti-Foxo antibody. **a**–**a''** Endogenous Foxo is mostly found in the cytoplasm. **b**–**b''** Tctp knockdown increases Foxo levels in the cytoplasm. For Tctp knockdown, S2 cells were treated with Tctp dsRNA twice a day for 3 days. Scale bar, 10 μm. **c** Effects of *Tctp* RNAi on the level of cytoplasmic Foxo in salivary glands. Foxo is undetectable in two control strains (*AB1/*+ and *AB1* > *GFP*) but is increased by *Tctp* RNAi (*AB1* > *Tctp i*). Asterisk indicates a non-specific band. **d** Effects of Tctp knockdown on the Foxo level in S2 cells. The cytoplasmic extracts from S2 cells treated with *Tctp* dsRNA show an enhanced level of Foxo compared with untreated cells. **e** Effects of human TCTP knockdown on hFOXO1. Endogenous hTCTP was knocked down by siRNA treatment in HeLa cells for 72 h. Immunoblotting shows that *hTCTP* siRNA leads to an increase in the hFOXO1 level. β-Tubulin shows a loading control.

Our data indicated that Tctp is required for nuclear localization of Foxo in the salivary gland (Supplementary Fig. 4). Foxo was detected mainly in the cytoplasm in *Drosophila* S2 cells (Fig. 7a-a''). Consistent with the data in the salivary gland, cytoplasmic Foxo levels were increased in S2 cells treated with *Tctp dsRNA* (Fig. 7b-b''). We also tested the role of Tctp in Foxo localization using western blot experiments. Cytosolic extracts of salivary glands from wild-type and *UAS-GFP* control larvae showed low Foxo levels. In contrast, *Tctp* knockdown by *UAS-Tctp* RNAi increased the Foxo level (Fig. 7c). Consistent with the immunocytochemistry data (Fig. 7a–b''), *Tctp* knockdown in S2 cells also led to an increased level of Foxo protein compared with control cells (Fig. 7d).

**Fig. 8:**
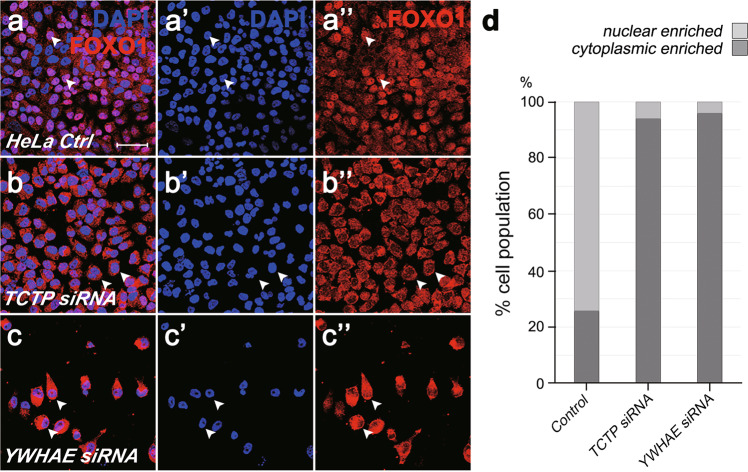
Knockdown of human TCTP or YWHAE increases cytoplasmic FOXO in HeLa cells. **a**–**c** HeLa cells were stained with DAPI and the anti-FOXO1 antibody. **a**–**a''** Control cells. Endogenous FOXO was mainly localized in the nucleus (white arrowheads). **b**–**b''** Cells with TCTP siRNA. Nuclear FOXO levels were significantly reduced by TCTP knockdown while cytoplasmic levels were increased. **c**–**c''** Cells with YWHAE siRNA. Cell numbers were decreased. Most cells show cytoplasmic enrichment of FOXO. Scale bar, 50 μm. **d** Quantification of data shown in (**a**–**c''**). Cells were scored based on the FOXO enrichment in the nucleus (light gray) or the cytoplasm (dark gray).

Next, we tested whether the role of Tctp is conserved in human cells. In HeLa cell extracts, the anti-human FOXO1 (hFOXO1) antibody detected a major band at about 85 kDa. After 72 h treatment with human *TCTP* (*hTCTP*) siRNA, there was a strong increase in the hFOXO1 protein (Fig. 7e). To examine the effects of *hTCTP* siRNA on the subcellular distribution of hFOXO1, we carried out immunocytochemical analysis in HeLa cells. In control cells, most of the cells (74 ± 1.4%; Fig. 8d) showed intense FOXO1 staining in the nucleus and the rest of the cells showed cytoplasmic or weak ubiquitous staining (Fig. 8a–a''). *hTCTP* knockdown resulted in a drastic change in the FOXO1 localization (Fig. 8b–b''). 94 ± 5.2% of *hTCTP*-depleted cells showed cytoplasmic enrichment of FOXO1 while reducing nuclear FOXO1 levels (Fig. 8d). We also checked the effects of silencing human *14-3-3ε* homolog *YWHAE*. Knockdown of YWHAE considerably decreased cell viability (approximately 40% of control HeLa cells). 96 ± 4.0% of survived *YWHAE*-depleted cells showed strong enrichment of FOXO1 in the cytoplasm (Fig. 8c–c'', d). These data indicate that hTCTP and YWHAE play similar roles in the nuclear localization of FOXO1 as their *Drosophila* homologs in the salivary gland.

Overall, these results suggest that Tctp is involved in regulating the level and the localization of Foxo. In addition, the role of Tctp in the Foxo regulation may be conserved in human cells.

## Discussion

We have addressed the roles of Tctp and 14-3-3 isoforms in regulating Foxo functions in the developing eye and the salivary gland. Our data suggest that Tctp and 14-3-3ε play roles in tissue growth by regulating the level and the localization of Foxo protein.

### Role of Tctp in Foxo regulation during eye development

We find that the eye phenotype of *ey-Gal4*-driven Foxo overexpression in undifferentiated cells of the eye disc is suppressed by Tctp overexpression (Figs. 1 and 2). Knockdown of *Tctp* also increases the endogenous Foxo level in parallel with reduction of the eye field (Fig. 3), which is consistent with a reduction of eye disc size by Foxo overexpression (Fig. 2). In addition, Tctp overexpression reduces Foxo levels in the eye disc (Fig. 2), supporting the negative regulation of the Foxo level by Tctp.

Because Tctp overexpression reduces the level of exogenous Foxo expressed from a transgene, Tctp may downregulate Foxo by a post-transcriptional mechanism rather than by affecting *foxo* transcription. Tctp and 14-3-3 physically interact for organ growth [7] and that 14-3-3 proteins coimmunoprecipitate with Foxo [10]. Hence, the interaction between Tctp and 14-3-3ε proteins may be necessary for the proper regulation of Foxo. The increased Foxo levels by *Tctp* knockdown might be partly due to the abnormal localization of Foxo. However, we were unable to reliably detect mislocalized Foxo in the retinal cells of the eye disc. High-resolution imaging analysis would be helpful in determining the effects of Tctp and 14-3-3ε on Foxo localization.

### Role of Tctp and 14-3-3ε in Foxo localization in the salivary gland

We used large epithelial cells of the salivary gland as an alternative approach to examine the effects of Tctp on the Foxo localization. As in the eye, we find that Tctp and 14-3-3 are required for the growth of the salivary gland. 14-3-3ε and 14-3-3ζ have redundant functions in imaginal discs [7], but have distinct roles in other tissues [9, 28]. Our data show that 14-3-3ε, but not 14-3-3ζ, is essential for cell growth in salivary glands, providing additional evidence for isoform-specific functions of 14-3-3 proteins. Although 14-3-3ζ is not essential for the growth of the salivary gland, the abnormal Foxo distribution resulting from *Tctp* RNAi is partially suppressed by *14-3-3ζ* knockdown (Supplementary Fig. 6). Since the loss of *14-3-3ε* is compensated by upregulation of 14-3-3ζ in a tissue-specific manner [9], loss of *14-3-3ζ* may lead to an upregulation of 14-3-3ε in the salivary gland, thus leading to the suppression of *Tctp* RNAi phenotype.

Knockdown of *Tctp* or *14-3-3ε* shows similar phenotypes in the salivary gland such as reduced cell size and upregulation of cytoplasmic Foxo levels. Because the phenotype of double knockdown of *Tctp* and *14-3-3ε* is comparable to that of *14-3-3ε* RNAi alone, these two genes might be involved in the same pathway. Based on the direct interaction between Tctp and 14-3-3 [7] and co-immunoprecipitation of 14-3-3 and Foxo [10], 14-3-3ε and Tctp may regulate nuclear localization of Foxo in a protein complex, although it is unknown whether they directly interact with Foxo. Another interesting question is whether Tctp and 14-3-3ε are also involved in modulating the level of Foxo. *Tctp* knockdown increases Foxo levels in the eye disc (Fig. 3). Similarly, Foxo levels are increased in Tctp-depleted S2 cells (Fig. 7). Thus, it appears that Tctp is not only involved in the nuclear Foxo localization but also in the maintenance of the cytoplasmic Foxo protein level.

14-3-3ε overexpression partially suppresses the effects of Foxo overexpression in the eye [10] but not in the salivary gland (Supplementary Fig. 3). Interestingly, we notice that overexpression of 14-3-3ε weakly inhibits the growth of salivary glands (Supplementary Fig. 2) while it has no impact in eye/wing imaginal discs [7]. The failure to suppress the Foxo effect by 14-3-3ε overexpression may be related to the negative effects of 14-3-3ε overexpression in the salivary gland. It has been shown that knockdown or overexpression of mammalian 14-3-3γ results in similar phenotypes in neuronal cell migration [29]. These results suggest that the level of some 14-3-3 isoforms must be tightly regulated to function properly in vivo.

The main role of Foxo family proteins is to activate transcription [28, 30, 31]. However, our data show that the exogenous Foxo protein overexpressed in the salivary gland, which causes growth defects in the gland, is enriched in the cytoplasm (Fig. 6). Hence, Foxo-mediated growth defects in the salivary gland seem to be related to the cytoplasmic accumulation of Foxo. Furthermore, knockdown of *Tctp* or *14-3-3ε* elevates the endogenous cytoplasmic Foxo level while reducing the nuclear Foxo level (Supplementary Fig. 4 and 5). These results indicate that Tctp and 14-3-3ε are required to antagonize cytoplasmic accumulation of Foxo by promoting its nuclear localization. Furthermore, Tctp overexpression increases the nuclear Foxo level in the Foxo-overexpressing salivary gland (Fig. 6c), indicating that Tctp is required and sufficient for Foxo nuclear localization. It is also worth noting that loss of *Tctp* or *14-3-3ε* results in a reduction of CycE and BrdU labeling in the nucleus (Fig. 5). Thus, Tctp and 14-3-3ε are required for nuclear localization of CycE as well as Foxo for endoreplication in the larval salivary gland.

### Regulation of human FOXO localization by TCTP

The eye phenotype of *Tctp* loss-of-function can be rescued by hTCTP, suggesting a conserved role of *Drosophila* Tctp and hTCTP [18]. We found that overexpression of *Drosophila* Tctp partially rescues Foxo mislocalization in the salivary gland (Fig. 6) but hTCTP does not (Supplementary Fig. 3f–f'), suggesting that hTCTP may act differently from *Drosophila* Tctp in the salivary gland. Indeed, unlike Tctp, hTCTP overexpression causes the ubiquitous distribution of endogenous Foxo in the salivary gland (Supplementary Fig. 3e–e'). Hence, hTCTP overexpression may not provide a proper condition to suppress the Foxo overexpression phenotype in the salivary gland. Although hTCTP transgene did not mimic the effects of *Drosophila* Tctp in the salivary gland, we observed that endogenous human FOXO (hFOXO) is enriched in the nucleus of HeLa cells, like *Drosophila* Foxo in the salivary gland. Furthermore, knockdown of *hTCTP* or *YWHAE* causes cytoplasmic localization of hFOXO (Fig. 8). Hence, as in the *Drosophila* salivary gland, hTCTP and YWHAE are required for nuclear enrichment of hFOXO in human cells.

Phosphorylation of FOXO3a upon insulin signaling leads to the export of nuclear FOXO, thus reducing the transcriptional FOXO function [13]. In *Drosophila*, it was proposed that loss of *14-3-3* allows a nuclear entry of Foxo, thereby activating transcription to promote apoptosis and repress cell growth [10]. Our analysis suggests that growth inhibition in the salivary gland might be due to Foxo accumulation in the cytoplasm rather than the nucleus. It remains to be studied whether the proposed effect of cytoplasmic Foxo is a unique phenomenon in the salivary gland. Nonetheless, our finding of similar cytoplasmic enrichment of hFOXO by the reduction of hTCTP or YWHAE implies that cytoplasmic FOXO may also have functional significance in human cells.

In addition to the FOXO roles for transcription, FOXO has transcription-independent functions in the cytoplasm. For example, cytosolic FOXO1 acts as a tumor suppressor or stress sensor by inducing autophagy in response to stress [31, 32]. It is an interesting question whether the increased cytoplasmic Foxo inhibits cell growth through transcription-independent mechanisms in developing mammalian tissues and organs.

## Materials and methods

### Fly genetics

The Gal4 drivers, *ey-Gal4* (5534) and *AB1-Gal4* (1824) were obtained from Bloomington *Drosophila* Stock Center (BDSC). *UAS-RFP* (30556 on the second or 31417 on the third chromosome) (BDSC) was used for marking the Gal4 expression region. *UAS-Tctp*, *UAS-Tctp RNAi, and UAS-hTCTP* lines were as described [18], and another RNAi line (45532) was from Vienna *Drosophila* Resource Center (VDRC). For 14-3-3 knockdown, *UAS-14-3-3ε RNAi* (34884, BDSC and 31196R-3, National Institute of Genetics, Japan) and *UAS-14-3-3ζ RNAi* (VDRC v48725, BDSC 41878, and BDSC 28327) were used. *UAS-myc-14-3-3ε* was a gift from Dr. Efthimios Skoulakis. For double knockdown of *Tctp* and *14-3-3*, *ey* > *Tctp RNAi/CyO* recombinants were crossed with *14-3-3ε* RNAi or *14-3-3ζ* RNAi lines. *UAS-foxo*^*ORF*^ (F000143, FLYORF) and *UAS-foxo* (9575, BDSC) were used for dFoxo overexpression. The cross of *UAS-foxo* (9575, BDSC) was carried out at 18 ^°^C to reduce the severe phenotype of small salivary glands. All other flies were grown at 25^o^C.

### Immunohistochemistry

*Drosophila* imaginal discs were dissected from third instar larvae and stained as described [33, 34]. S2 cells were cultured on a slide glass chamber and fixed with 4% paraformaldehyde. After fixation, samples were immersed in blocking solution for 1 h at RT. The tissues or cells were then incubated at 4 ^°^C overnight sequentially in the primary and secondary antibody mix diluted in washing buffer (50 mM Tris pH6.8; 150 mM NaCl; 0.5% Igepal CA-630, Sigma-Aldrich; 1 mg/ml bovine serum albumin; 0.02% sodium azide). Finally, samples were stained with DAPI and mounted with a mounting medium (Vectashield). The following primary antibodies were used for immunohistochemistry: rabbit anti-Foxo (1:200; THU-A-DFOXO, Cosmo Bio) and mouse anti-Arm (1:100, N2 7A1, Developmental Studies Hybridoma Bank, DSHB). Stained images were acquired using Zeiss confocal microscope LSM 710. Analysis of cell number and size of salivary glands was performed using the Image J program, and the data were presented with standard deviation (SD). Statistical significance was determined by a *t*-test of Microsoft Excel.

For CycE immunostaining, late 3rd instar larvae were dissected in 1X PBS, then fixed in 1X PBS with 2% paraformaldehyde for 30 min. After washing three times 5 min each with 0.1% PBTr, the glands were incubated in 0.1% PBTr with rabbit anti-CycE (1:100, d-300, Santa Cruz Biotechnology) at 4 ^°^C overnight. Glands were blocked in 0.1% PBTr with 5% normal goat serum for 1 h before incubating with the secondary antibody of goat anti-rabbit Alexa 488 (1:500, Invitrogen A11008) for 2 h at RT. The samples were washed four times, 15 min each with 0.1% PBTr. DAPI (1:2000) and/or phalloidin (1:250) was added during the second wash.

### BrdU labeling

For BrdU labeling, third instar larvae were raised in food vials containing 0.25 mg/ml BrdU for 24 h before dissection. Wandering 3rd instar larvae were dissected for salivary glands in 1X PBS, then fixed in 1X PBS with 2% paraformaldehyde for 30 min. After washing three times 5 min each with 0.1% PBTr (1X PBS with 0.1% Triton X-100), glands were treated with 3 N HCl for 30 min to denature the BrdU-labeled DNA. The glands were neutralized by washing with 0.1% PBTr for three times, 5 min each before incubating in 0.1% PBTr with mouse anti-BrdU (1:50) at 4 ^°^C overnight. Glands were blocked in 0.1% PBTr with 5% normal goat serum for 1 h before incubating with the secondary antibody of goat anti-mouse Alexa 488 (1:500, Invitrogen A11001) for 2 h at RT. The samples were washed four times 15 min each with 0.1% PBTr. DAPI (1:2000) was added during the second wash.

### *Drosophila* S2 cell culture and double-strand RNA (dsRNA) treatment

*Drosophila* S2 (stock #6) was obtained from *Drosophila* Genomics Resource Center (DGRC). The cells were maintained with 10% artificial serum in M2 media (Sigma-Aldrich). To knock down endogenous Tctp in S2 cells, dsRNA was designed and synthesized in vitro using MEGAscript RNAi Kit (Ambion): *anti-Tctp* forward 5'-GAGATGTTTGCCGACACCTAC-AA and reverse 5'-GCCGTCGCAGTCCATAGATTC primers conjugated downstream of T7 promoter sequences according to the manufacturer’s instructions. S2 cells were treated twice with 40 nM *Tctp dsRNA* every 3 days and harvested on day 6.

### Immunoblot analysis

Late third instar larvae or S2 cells were lysed with lysis buffer (20 mM HEPES pH7.4, 70 mM KCl, 10 mM EDTA, 10 mM EGTA, 2 mM DTT, 1 mM PMSF, 0.1% Igepal CA-630, 16% Glycerol, and Roche EDTA-free Protease inhibitor cocktail) chilled on ice. The lysates were boiled with sample loading buffer at 95 ^°^C for 10 mins. The total protein samples were loaded on 7.5% or 10% SDS-PAGE gel and transferred to a nitrocellulose membrane. After blocking at RT, membranes were probed sequentially in primary antibody solution and HRP-conjugated secondary antibody solution diluted with 2% dry milk or 2% BSA in TBST (140 mM NaCl; 3 mM KCl; 25 mM Tris pH7.4; 0.1% Tween 20). Pierce ECL western blotting substrate (Thermo) was used to detect immunostained proteins. A cytosolic fraction from salivary glands was isolated as described [35]. Primary antibodies were rabbit anti-hFOXO1 (1:1000; A2934, Abclonal), rabbit anti-dFoxo (1:1000; ab195977, Abcam), rabbit anti-hTCTP (1:2000; ab37506, Abcam), rabbit anti-Tctp (1:2500) [18], mouse anti-βTub (1:5000; E7, DSHB), and rabbit anti-GFP (1:10000; ab290, Abcam).

### Mammalian cell culture, siRNA treatment, and immunostaining

The human HeLa cell line was cultured with high glucose DMEM, 10% fetal bovine serum, 1% penicillin/streptomycin at 37^o^C, 5% CO_2_ condition. The sense strand of siRNAs was synthesized using target sequences for hTCTP (5′-GGTAACATTGATGACTCGC-3') [36] and for YWHAE (5'-AAGCTGGCCGAGCAGGCTGAG-3'). Sense and anti-sense strands were synthesized from Bioneer. Cells were incubated for 3 days in the Opti-MEM media (Gibco) containing 100 nM siRNA with lipofectamine RNAiMAX reagent (Thermo Fisher Scientific).

For immunostaining of human cells, HeLa cells were seeded on cover glasses coated with 0.01% poly-L-lysine 24 h before siRNA treatment. Cells were treated with 100 nM hTCTP or YWHAE siRNA for 72 h and fixed in 4% paraformaldehyde. Fixed cells were in blocking solution (10% normal goat serum; 1% bovine serum albumin; 0.1% Tween 20; in 1X PBS) for 1 h, then incubated with rabbit anti-FOXO1 (1:100; A2934, Abclonal) diluted in washing buffer (0.5% bovine serum albumin; 0.05% Tween 20; in 1X PBS) followed by secondary antibody at 4 ^°^C overnight. Finally, cells were stained with DAPI.

## Supplementary information

Supplementary Info
