# Supplementary material for: Tctp regulates the level and localization of Foxo for cell growth in Drosophila
Source: Cell Death Discov. 2022 Mar 31;8:146. doi: 10.1038/s41420-022-00937-2 (PMC8971462; doi:10.1038/s41420-022-00937-2)

**Supplementary Information**

**Supplementary Figure 1.**

**Effects of independent RNAi lines for Tctp and 14-3-3 on the salivary gland
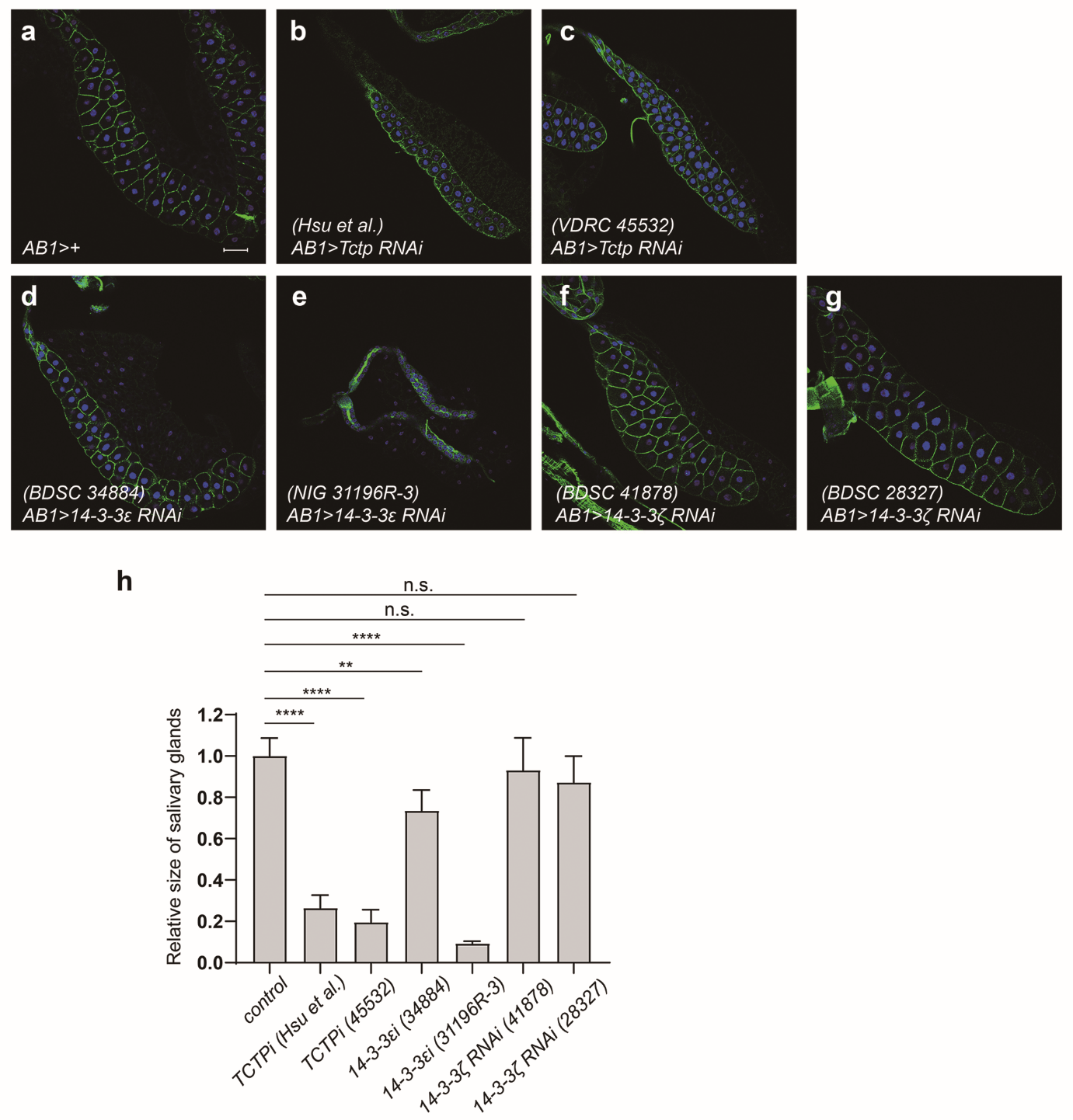
**

Salivary glands were stained with phalloidin (green) and DAPI (blue) to label cell boundaries and nuclei, respectively. *AB1-Gal4* was used to drive RNAi. **(a)** A control salivary gland. **(b-c)** Knockdown of *Tctp* using two RNAi lines from Hsu *et al*., 2007 (b) and VDRC 45532 (c). **(d-e)** Knockdown of *14-3-3ε* using two RNAi lines, BDSC 34884 (d) and NIG 31196R-3. **(f-g)** Knockdown of *14-3-3ζ* using two RNAi lines, BDSC 41878 (f) and BDSC 28327 (g). **(h)** Quantification of the salivary gland size shown in (a-g). The areas of salivary glands were measured using the Image J program and presented as ratios relative to the control size. Five salivary glands from five larvae for each genotype were measured. Standard deviations are indicated by error bars. Statistical significances are indicated by black asterisks (**p<0.01, ****p<0.0001, and n.s., not significant). Scale bar, 100µm.

**Supplementary Figure 2.**

**Effects of Tctp/14-3-3**ε **overexpression on Tctp/14-3-3**ε **RNAi phenotypes in the salivary gland**


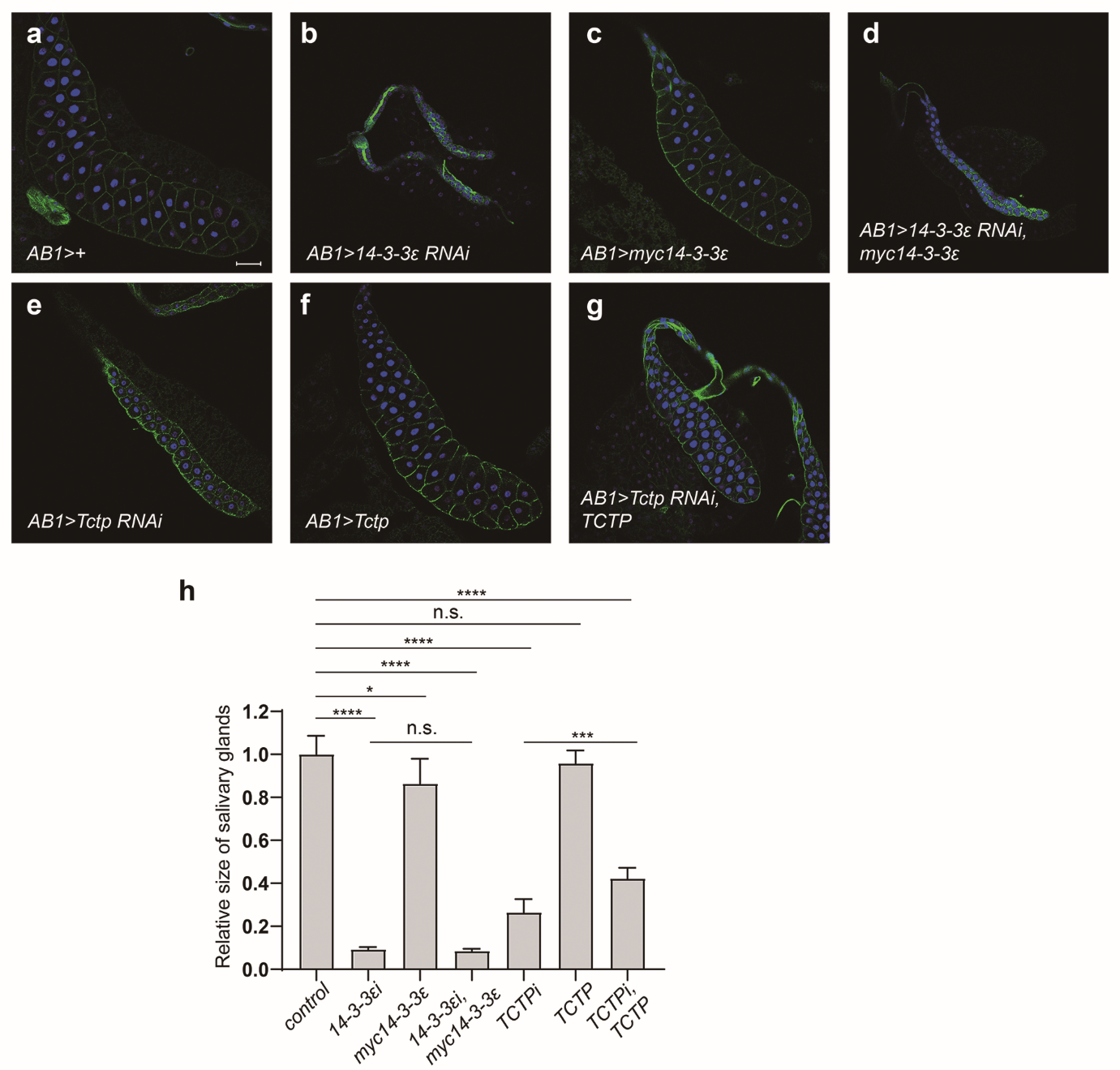
Salivary glands were stained with phalloidin (green) and DAPI (blue) to label the cell boundaries and nuclei, respectively. **(a)** Control salivary gland. **(b)** *14-3-3ε* RNAi results in a strong reduction of the salivary gland size. **(c)** Overexpression of 14-3-3ε shows a weak reduction of the salivary gland size. **(d)** Overexpression of 14-3-3ε has no significant effect in rescuing the *14-3-3ε* RNAi phenotype. **(e)** *Tctp* RNAi reduces the salivary gland size. **(f)** Overexpression of Tctp has no effect on the salivary gland size. **(g)** Overexpression of Tctp partially rescues the *Tctp* RNAi phenotype. **(h)** Quantification of the salivary gland size shown in (a-g). The areas of salivary glands were measured using the Image J program and presented as ratios relative to the control size. Five salivary glands from five larvae for each genotype were measured. Standard deviations are indicated by error bars. Statistical significances are indicated by black asterisks (*p<0.05, ***p<0.01, ****p<0.001, and n.s., not significant). Scale bar, 100µm.

**Supplementary Figure 3.**

**Foxo overexpression phenotype in the salivary gland is not rescued by overexpression of 14-3-3ε or human TCTP**

**
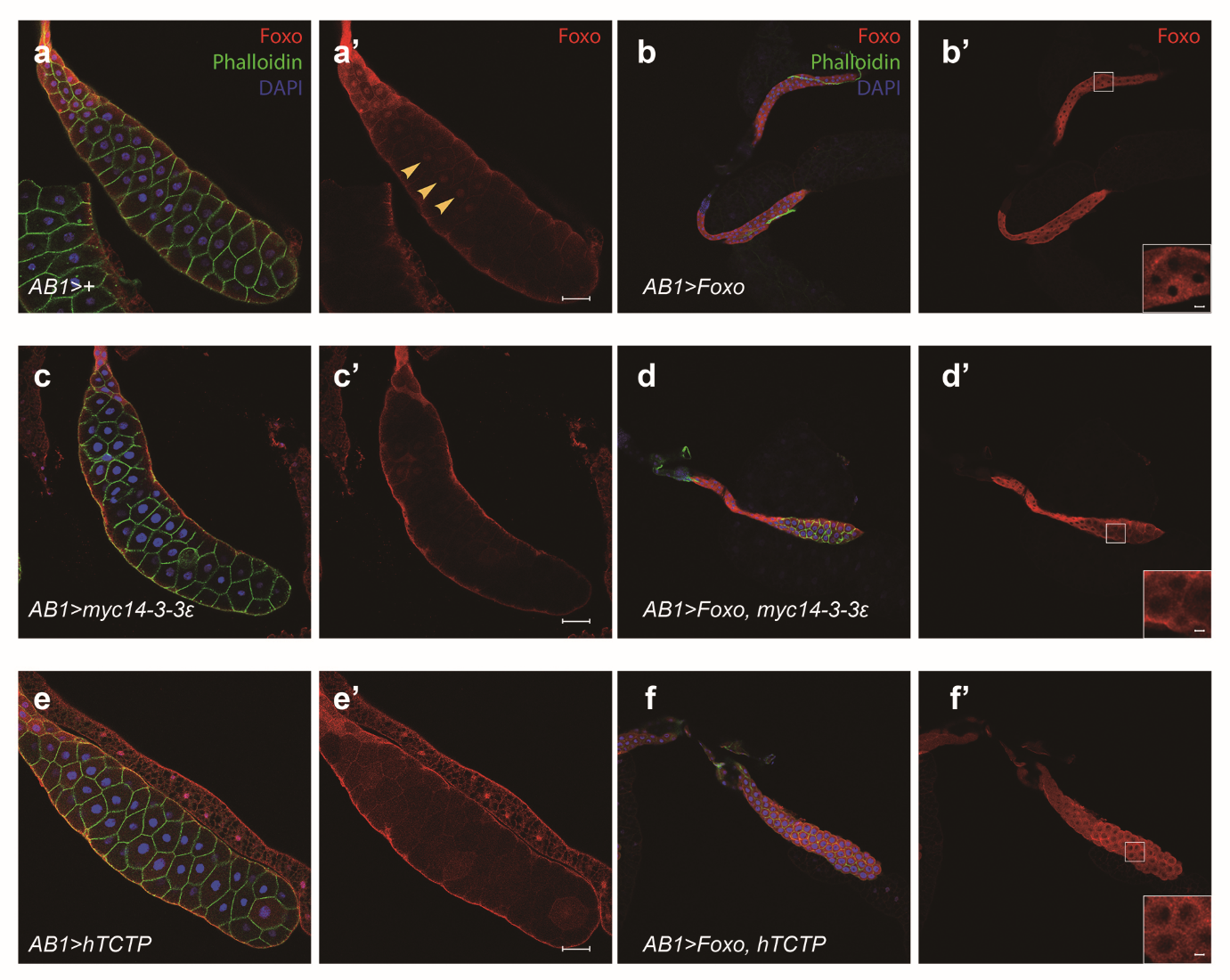
**Subcellular localization of Foxo is not rescued by overexpression of 14-3-3ε and hTCTP. Salivary glands were labeled with phalloidin (green), DAPI (blue), and anti-Foxo antibody (red). **(a)** Control salivary gland. Merged image (a) and the Foxo channel (a’) show a weak nuclear staining of endogenous Foxo (yellow arrowheads). **(b)** Effects of Foxo overexpression. Merged image (b) and the Foxo channel (b’) show a strong reduction of the salivary gland size and cytoplasmic enrichment of Foxo (inset). **(c)** Effects of 14-3-3ε overexpression. Merged image (c) and the Foxo channel (c’) show a mild reduction of the salivary gland size and a weaker Foxo staining than the control. **(d)** Co-overexpression of Foxo and 14-3-3ε. Merged image (d). 14-3-3ε overexpression does not alter the cytoplasmic localization of overexpressed Foxo (d’, inset). **(e-e’)** Effects of hTCTP overexpression. Merged image (e) and the Foxo channel (e’) show ubiquitous distribution of Foxo. **(f-f’)** Co-overexpression of Foxo and hTCTP. Merged image (f). hTCTP overexpression does not alter the cytoplasmic localization of overexpressed Foxo (f’, inset). Scale bar, 100µm.

**Supplementary Figure 4.**

**Tctp regulates Foxo distribution between nucleus and cytoplasm**


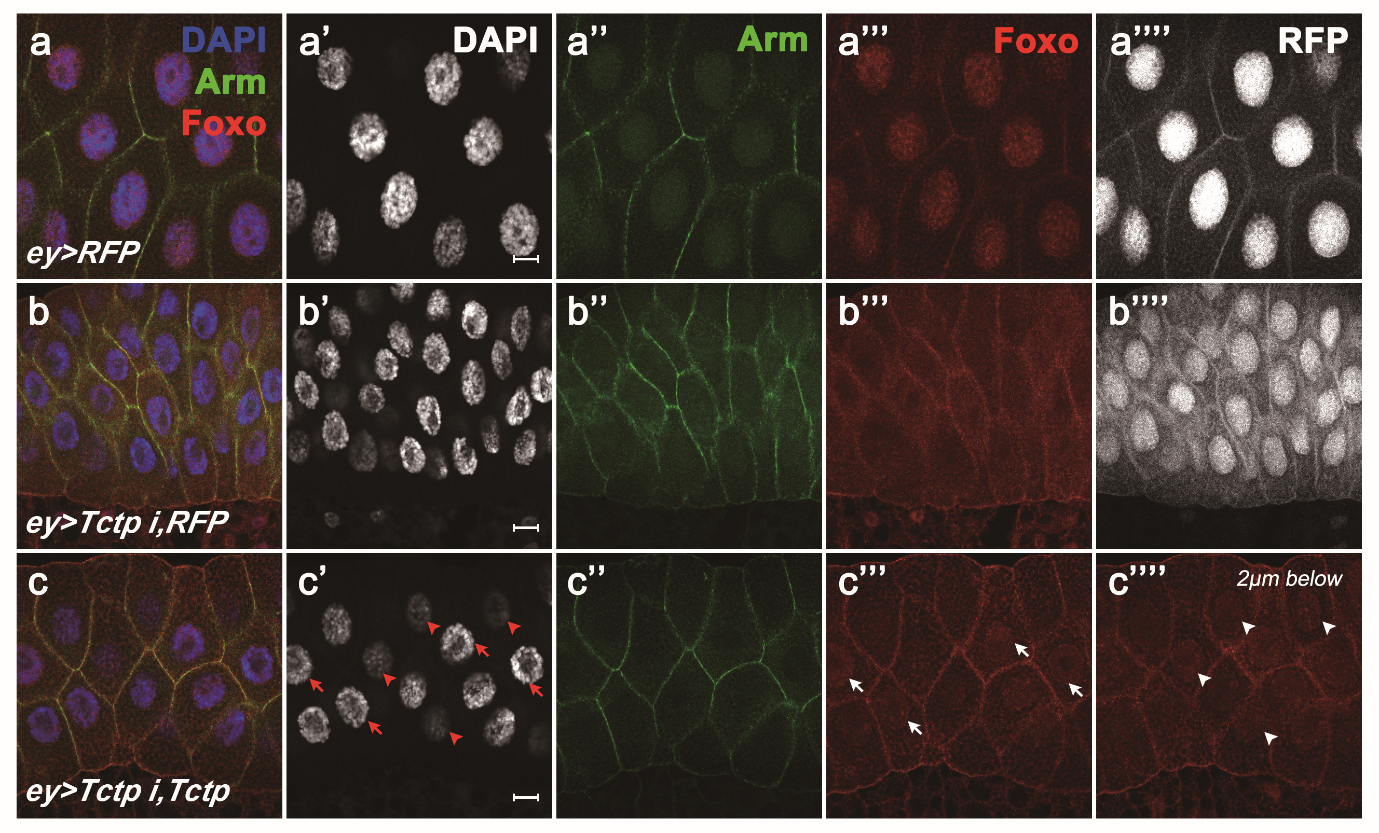
Salivary glands of late third instar larvae were dissected and stained with DAPI, anti-Arm, and anti-Foxo. **(a)** *ey>RFP* control. **(b)** *ey>Tctp* RNAi*, RFP*. Tctp-depleted cells are reduced in size (b”) and the level of nuclear Foxo (b”’). **(c)** *ey>Tctp* RNAi*, Tctp*. Cytoplasmic localization of Foxo caused by *Tctp* RNAi is rescued by overexpression of wild-type Tctp. Recovery of nuclear Foxo is shown at two different focal planes (white arrows and arrowheads in c’” and c””, respectively. DAPI staining of these nuclei are indicated by red arrows and arrowheads in c’). Scale bar, 20μm.

**Supplementary Figure 5.**

**Cytosolic Foxo levels are increased by knockdown of 14-3-3ε but not 14-3-3ζ**


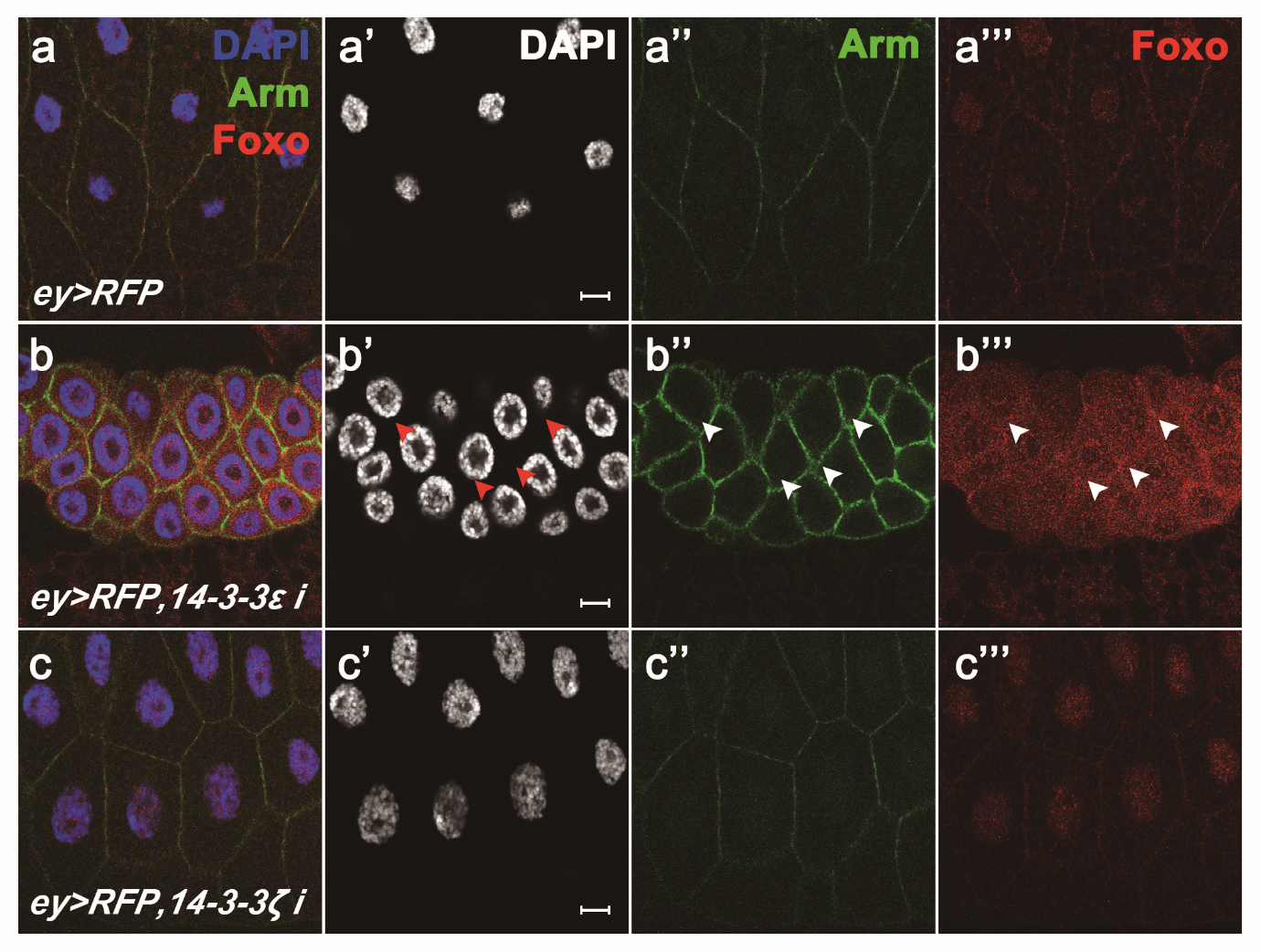
Effects of 14-3-3 knockdown on the endogenous Foxo. Salivary glands from third instar larvae were stained for Arm and Foxo. **(a)** *ey>RFP* control. **(b)** *ey>RFP,14-3-3ε* RNAi. 14-3-3ε knockdown increases endogenous Foxo in the cytoplasm (b”’, white arrowheads). The same positions are indicated in b’ and b” by red and white arrowheads, respectively. **(c)** *ey>RFP,14-3-3ζ* RNAi. Knockdown of 14-3-3ζ has no significant changes in the Foxo staining (c”’). Scale bar, 20μm.

**Supplementary Figure 6.**

**Foxo mislocalization due to Tctp knockdown is suppressed by *14-3-3ζ* RNAi**


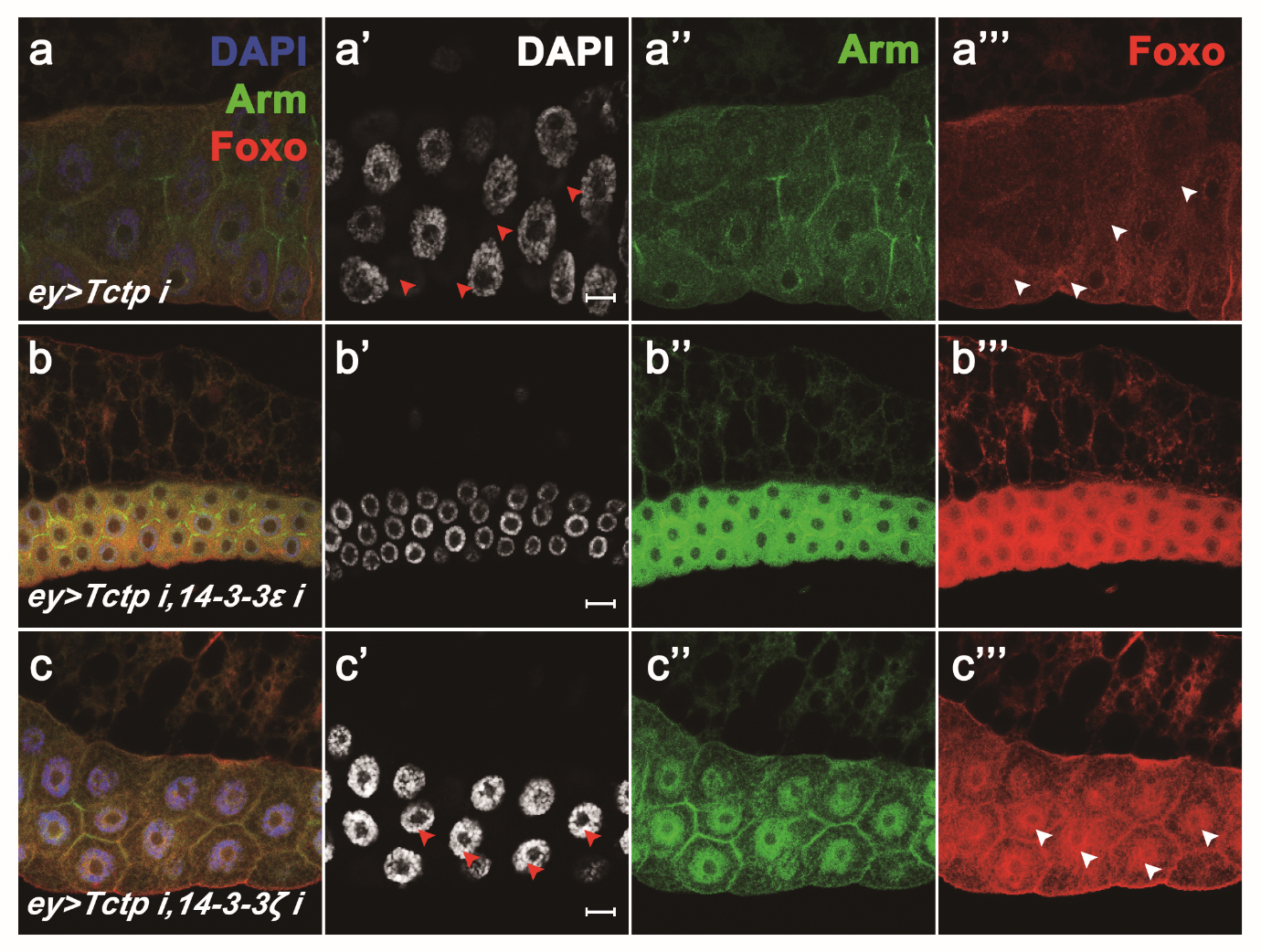
Effects of double knockdown of each 14-3-3 isoform and Tctp on the endogenous Foxo localization. Salivary glands were stained with DAPI and antibodies for Arm and Foxo. **(a)** *ey>Tctp RNAi* was used as a control that shows cytoplasmic localization of Foxo (white arrowheads in a”’). **(b)** *ey>Tctp RNAi,14-3-3ε RNAi*. Double knockdown of *Tctp* and *14-3-3ε* results in glands with a strongly reduced size and enriched Foxo in the cytoplasm. **(c)** *ey>Tctp RNAi,14-3-3ζ RNAi*. Knockdown of *14-3-3ζ* suppresses the effect of *Tctp* RNAi, resulting in increased levels of nuclear Foxo (arrowheads with red in c’ and white in c”’). Scale bar, 20μm.

- Original WB film


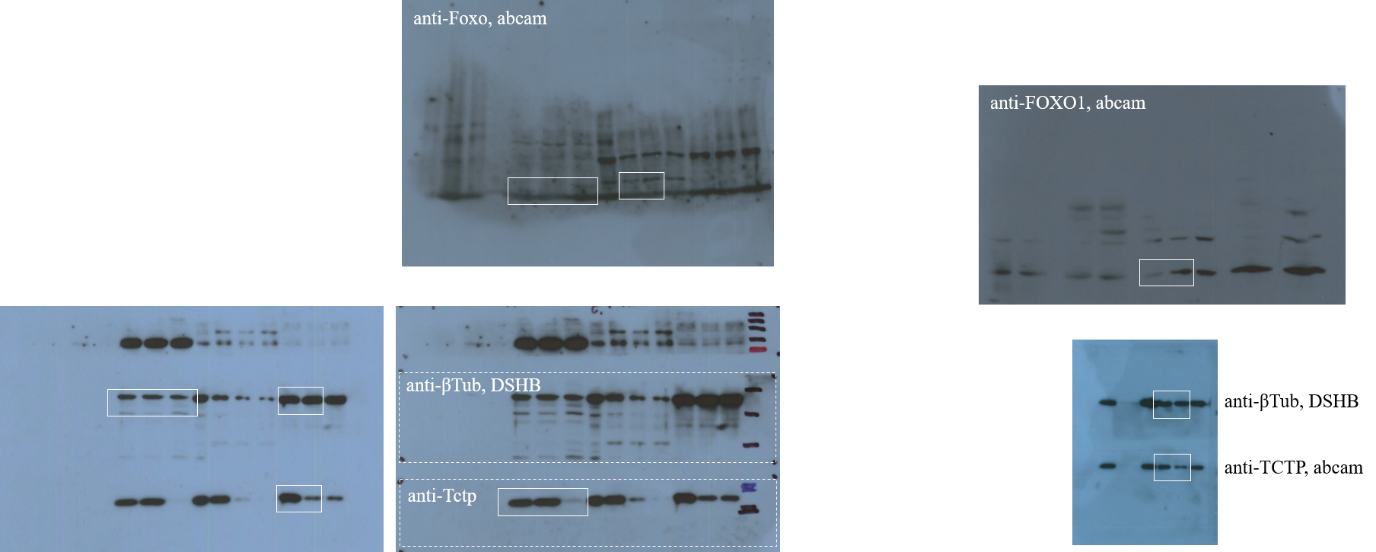

Supplement: Supplementary file 1 — Supplementary Info [file 41420_2022_937_MOESM1_ESM.docx]
